# The Association between TIF1 Family Members and Cancer Stemness in Solid Tumors

**DOI:** 10.3390/cancers13071528

**Published:** 2021-03-26

**Authors:** Patrycja Czerwinska, Nikola Agata Wlodarczyk, Anna Maria Jaworska, Andrzej Adam Mackiewicz

**Affiliations:** 1 Department of Cancer Immunology, Chair of Medical Biotechnology, Poznan University of Medical Sciences, 15 Garbary St., 61-866 Poznan, Poland; nikola.wlodarczyk95@wp.pl (N.A.W.); am.jaworska96@gmail.com (A.M.J.); 2 Department of Diagnostics and Cancer Immunology, Greater Poland Cancer Centre,15 Garbary St., 61-866 Poznan, Poland

**Keywords:** cancer stemness, stemness score, TIF1, TRIM28, TCGA

## Abstract

**Simple Summary:**

Stem cell-associated molecular features of solid tumors, collectively known as cancer stemness, are of great importance in the development, progression, and reoccurrence of cancer. Transcriptional and epigenetic dysregulation is significantly associated with cancer stemness. Here, we investigated the association between the Transcriptional Intermediary Factor 1 (TIF1) family members and cancer stemness in solid tumors. We aimed to evaluate the potential value of TIF1 members in predicting a stem-like cancer phenotype. Our results indicate that only TIF1β (also known as Tripartite Motif protein 28, TRIM28) high expression is consequently associated with a “stemness high” phenotype, regardless of the tumor type, resulting in a worse prognosis for cancer patients. The oncogenic signature of TRIM28^HIGH^ tumors significantly reflects the enrichment of “stemness high” cancers with targets for c-Myc (MYC Proto-Oncogene). TRIM28-associated gene expression profiles are also robustly enriched with stemness markers. Our results demonstrate that the association between high TRIM28 expression and an enriched cancer stem cell-like phenotype is a common phenomenon across solid tumors.

**Abstract:**

Cancer progression entails a gradual loss of a differentiated phenotype in parallel with the acquisition of stem cell-like features. Cancer de-differentiation and the acquisition of stemness features are mediated by the transcriptional and epigenetic dysregulation of cancer cells. Here, using publicly available data from The Cancer Genome Atlas (TCGA) and Gene Expression Omnibus (GEO) databases and harnessing several bioinformatic tools, we characterized the association between Transcriptional Intermediary Factor 1 (TIF1) family members and cancer stemness in 27 distinct types of solid tumors. We aimed to define the prognostic value for TIF1 members in predicting a stem cell-like cancer phenotype and patient outcome. Our results demonstrate that high expression of only one member of the TIF1 family, namely TIF1β (also known as Tripartite Motif protein 28, TRIM28) is consequently associated with enriched cancer stemness across the tested solid tumor types, resulting in a worse prognosis for cancer patients. TRIM28 is highly expressed in higher grade tumors that exhibit stem cell-like traits. In contrast to other TIF1 members, only TIF1β/TRIM28-associated gene expression profiles were robustly enriched with stemness markers regardless of the tumor type. Our work demonstrates that TIF1 family members exhibit distinct expression patterns in stem cell-like tumors, despite their structural and functional similarity. Among other TIF1 members, only TRIM28 might serve as a marker of cancer stemness features.

## 1. Introduction

Cancer progression entails a gradual loss of a differentiated phenotype in parallel with the acquisition of stem cell-like features. Cancer cells that acquire stem cell-like traits are able to self-renew and to differentiate into bulk tumor cells [1,2]. De-differentiated primary tumors more frequently result in metastatic spread to distant organs, facilitating disease progression and a poor prognosis. These features, collectively known as cancer stemness, were recognized as valuable predictive or prognostic characteristics [3]. The molecular signatures capable of grading cancer stemness represent an essential step in designing novel therapeutic regimens that eventually may target the cancer stem cell population [4,5,6].

Recently, Malta et al. harnessed publicly available molecular profiles of distinct stem cell populations to quantify tumor stemness [7]. Using a machine learning algorithm, they developed a transcriptome-based stemness index (mRNA-SI) and demonstrated that higher values of the mRNA-SI were significantly associated with known biological processes active in cancer stem cells (CSCs) and with a higher de-differentiation status, as reflected in the histopathological grade. As presented previously, histologically poorly-differentiated tumors show preferential overexpression of genes normally enriched in embryonic stem cells (ESC) [4]. The ESC transcriptional program is frequently activated in diverse human epithelial cancers, suggesting its versatility in acquiring a cancer de-differentiation phenotype regardless of the tumor type. Reactivation of the ESC-like program in cancer strongly predicts metastatic potential and patient death [5,6].

Cell de-differentiation and the acquisition of stemness features is mediated by the transcriptional and epigenetic dysregulation of cancer cells [8]. Among other epigenetic factors, we focused on Tripartite Motif protein 24 TRIM24 (also known as Transcriptional Intermediary Factor 1α, TIF1α), TRIM28 (TIF1β), TRIM33 (TIF1γ), and TRIM66 (TIF1δ) proteins that comprise the Transcriptional Intermediary Factor 1 (TIF1) family of chromatin-binding proteins [9,10]. TIF1 family members act by remodeling chromatin templates and altering the activity of underlying transcriptional mechanisms. All four TIF1 proteins belong to a subfamily of the large, highly conserved tripartite-motif (TRIM) family of E3 ligases and are characterized by a similar structure. At the N-terminus, TIF1 members possess a RING finger/B-boxes/coiled coil (RBCC) motif, also known as the TRIM motif, which is made up of a Really Interesting New Gene (RING) domain (all except TRIM66), two B-boxes (B1 and B2), and a coiled-coil (CC) domain. The C-terminal plant homeodomain (PHD) finger and bromodomain (BROMO) unit is present in all TIF1 members and has been demonstrated to be indispensable for transcriptional repression through epigenetic mechanisms. TIF1 proteins can directly interact with modified histones via the PHD–BROMO unit, robustly contributing to the maintenance of genome stability [9,10].

TIF1 members are aberrantly expressed or mutated in multiple cancer types; however, their role in cancer stem cells is still not fully understood. The overexpression of TRIM24 is predominantly associated with cancer progression, inferring an oncogenic function [11,12,13,14,15]. TRIM24 was also identified as necessary for maintaining the undifferentiated state of totipotent cells during development [16,17,18]. Like TRIM24, TRIM28 is typically considered as an oncogene. TRIM28 expression is higher in tumor tissue when compared to adjacent healthy tissue in many distinct tumor types [19,20,21,22,23,24,25]. Previous reports demonstrate a fundamental role for TRIM28 in normal stem cell self-renewal, mediating repression of differentiation genes and inducing the expression of stemness markers [26,27]. TRIM28 was shown to be necessary for the acquisition of a stem cell-like phenotype by cancer cells. The downregulation of TRIM28 expression results in a loss of the stem cell-like phenotype in melanoma and breast cancer [20,28], although the TRIM28-mediated stemness-high tumor phenotype might be a universal phenomenon across distinct cancer types.

In contrast to the other TIF1 family members, TRIM33 has predominantly been identified as a tumor suppressor [29,30,31,32], although several studies imply its engagement in promoting cancer progression [33,34]. It was previously demonstrated that TRIM33 downregulation does not affect stem cell maintenance, but instead, it alters the cell differentiation process [35,36]. As for TRIM66, its role in cancer progression still remains largely unknown, although a few studies suggest that TRIM66 acts as an oncogene [37,38,39].

Here, we analyzed the association between the expression of TIF1 family members and cancer stemness across solid tumor types from The Cancer Genome Atlas (TCGA) database. We used TCGA and Gene Expression Omnibus (GEO) transcriptomic data to verify the relationship between the expression of TIF1 members and the level of cancer de-differentiation as assessed by previously reported stemness scores or signatures. We demonstrated that regardless of the tumor type, only high TRIM28 expression consequently corresponds to higher tumor stemness, while high TRIM66 expression is negatively associated with tumor stemness. Similarly, the transcriptome profiles of TRIM28^HIGH^ or TRIM66^HIGH^ tumors are significantly enriched or depleted with stemness markers, respectively, as demonstrated by the gene set enrichment analysis (GSEA). Higher grade tumors exhibit significantly elevated TRIM28 expression (with no change in other TIF1 members). Moreover, in many tumor types, “stemness high” tumors are significantly associated with a worse prognosis. Among other TIF1 family members, only high TRIM28 expression might serve as a marker for stemness-associated traits of solid tumors.

Our data demonstrate that the previously reported involvement of TRIM28 in the regulation of a stem-cell like phenotype in melanoma and breast cancer is a common phenomenon across distinct types of solid tumors. However, further studies are needed to define the exact mechanism(s) of TRIM28-mediated stemness acquisition and to verify its universality regardless of tumor type.

## 2. Materials and Methods

### 2.1. The TCGA Genomic and Clinical Data

In the current study, we used publicly available data for 27 solid TCGA tumors from cBioportal (www.cbioportal.org, accessed on 10 August 2020) (Table S1) [40,41] and the R2 Genomics Analysis and Visualization Platform (http://r2.amc.nl, accessed on 12 October 2020). We analyzed TCGA tumor types: (i) with more than 50 samples collected, and (ii) with survival data available (tumor types that were excluded: Cholangiocarcinoma (CHOL), Pheochromocytoma and Paraganglioma (PCPG), and Uterine Carcinosarcoma (UCS)). All data are available online, and access is unrestricted and does not require patient consent or other permissions. The use of the data does not violate the rights of any person or any institution.

### 2.2. Transcriptomic Data

The RNA sequencing-based mRNA expression data were directly downloaded from cBioportal. RNASeq V2 data from TCGA is processed and normalized using RSEM [42]. Specifically, the RNASeq V2 data in cBioPortal corresponds to the rsem.genes.normalized_results file from TCGA. Spearman’s correlation was used for the detection of co-expressed genes with a *p*-value < 0.05 and an False Discovery Rate (FDR) < 0.01 as cut-offs. Differentially expressed genes (DEGs) were cut off at *p*-value < 0.05 and FDR < 0.05.

### 2.3. The Human Protein Atlas

The representative results of immunohistochemistry staining of Prostate Adenocarcinoma (PRAD) samples with anti-TRIM24 (antibody name: HPA043495), anti-TRIM28 (HPA064033), anti-TRIM33 (HPA004345), and anti-TRIM66 (HPA027420) antibodies were downloaded from the Human Protein Atlas database (www.proteinatlas.org, accessed on 10 October 2020) [43]. For the samples presented in this study: TRIM24, low grade: Patient id (Pid)525, high grade: Pid3965; TRIM28, low grade: Pid3951, high grade: Pid4365; TRIM33, low grade: Pid525, high grade: Pid250; TRIM66, low grade: Pid3566, high grade: Pid3488.

### 2.4. Stemness-Associated Scores

The mRNA-SI stemness score [7] and other stemness signatures (Ben-Porath_ES_core, Wong_ESC_core, and Bhattacharya) used in this study were previously described [4,5,6]. When discriminating the all-score stemness high cohorts, only the samples that exhibited values above the average for each tested stemness score were defined as stemness^HIGH^ samples (4-score stemness^HIGH^). In the tumor types with the negative correlation between the stemness scores, we used only positively correlated scores to determine the stemness^HIGH^ cohort.

### 2.5. The Gene Set Enrichment Analysis

The gene set enrichment analysis (GSEA, http://www.broad.mit.edu/gsea/index.html, accessed on 31 August 2020) [44] was used to detect the coordinated expression of a priori defined groups of genes within the tested samples. Gene sets are available from the Molecular Signatures Database (MSigDB, http://www.broad.mit.edu/gsea/.msigdb/msigdb_index.html, accessed on 31 August 2020). All significantly correlated genes (previously ranked based on their Spearman’s correlation coefficient ® value) were imported to the GSEA. The GSEA was run according to the default parameters: each probe set was collapsed into a single gene vector (identified by its Human Genome Organisation (HUGO) gene symbol), permutation number = 1000, and permutation type = “gene-sets.” The FDR (<0.01) was used to correct for multiple comparisons and gene set sizes.

### 2.6. Validation Sets

Additional datasets used in this study (Table S2) [45,46,47,48,49,50,51,52,53] were obtained from the R2 Genomics Analysis and Visualization Platform. All datasets were analyzed online using the R2 Platform (http://r2.amc.nl, accessed on 10 October 2020) to find genes that correlate with TRIM24, TRIM28, TRIM33, or TRIM66 expression. All data are freely available online, and access is unrestricted and does not require patient consent or other permissions.

### 2.7. Statistical Analyses

Statistical analyses were carried out with GraphPad Prism 8.0 software (GraphPad Software, Inc., La Jolla, CA, USA). Multiple comparisons were performed with the ANOVA test. The correlation between two variables was assessed with Spearman’s rank correlation coefficient (r).

Survival analysis was performed according to the Kaplan–Meier analysis and log rank test. The overall survival (OS) was defined as the time between the date of surgery and date of death or the date of the last follow-up.

## 3. Results

### 3.1. TIF1 Family Members Are Differentially Expressed across Solid Tumors

**Figure 1:**
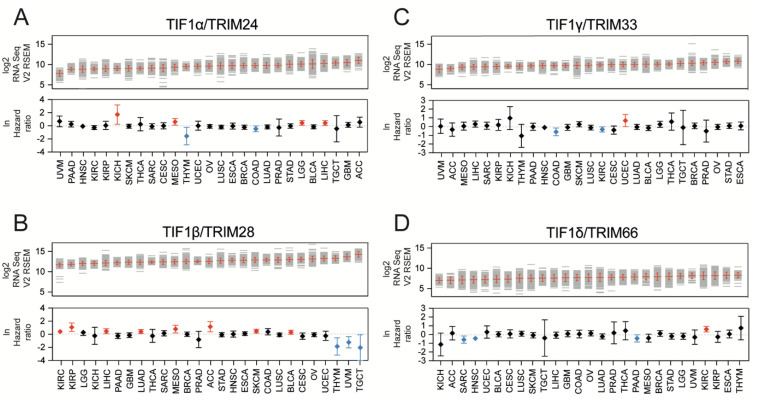
The expression of Transcriptional Intermediary Factor 1 (TIF1) family members across distinct solid tumor types. The expression of (**A**) Tripartite Motif protein 24 (TRIM24), (**B**) TRIM28, (**C**) TRIM33, and (**D**) TRIM66 in 27 solid tumor types based on RNA Seq RSEM V2 data from The Cancer Genome Atlas database (TCGA) (top panel). Each grey line represents a single tumor sample. The mean value and standard deviation (SD) within tumor type are marked with red. The bottom panel presents the hazard ratio (lnHR) of death for patients with high expression of specific TIF1 family members (with the mean as a cut-off). Red and blue denotes statistically significantly higher or lower hazard ratios, respectively. The HRs for individual tumor types are given with 95% confidence intervals (CIs). Tumor types: ACC—adrenocortical carcinoma; BLCA—bladder urothelial carcinoma; BRCA—breast invasive carcinoma; CESC—cervical squamous cell carcinoma and endocervical adenocarcinoma; COAD—colorectal adenocarcinoma; ESCA—esophageal carcinoma; GBM—glioblastoma multiforme; HNSC—head and neck squamous cell carcinoma; KICH—kidney chromophobe; KIRC—kidney renal clear cell carcinoma; KIRP—kidney renal papillary cell carcinoma; LGG—brain lower grade glioma; LIHC—liver hepatocellular carcinoma; LUAD—lung adenocarcinoma; LUSC—lung squamous cell carcinoma; MESO—mesothelioma; OV—ovarian serous cystadenocarcinoma; PAAD—pancreatic adenocarcinoma; PRAD—prostate adenocarcinoma; SARC—sarcoma; SKCM—skin cutaneous melanoma; STAD—stomach adenocarcinoma; TGCT—testicular germ cell tumor; THCA—thyroid carcinoma; THYM—thymoma; UCEC—uterine corpus endometrial carcinoma; UVM—uveal melanoma.

Using the cBioportal data, we analyzed the expression of four TIF1 family members, namely TIF1α/TRIM24, TIF1β/TRIM28, TIF1γ/TRIM33, and TIF1δ/TRIM66, across 27 solid tumor types to determine their association with patient survival. The mean values of the expression of TIF1 members in all tested tumor types are presented in Table S1. The abbreviations of the TCGA tumor types are explained in Table S1 and in the legend of Figure 1.

Using the mean expression value as a cut-off, we observed that higher TRIM24 expression is significantly associated with worse survival for Kidney Chromophobe (KICH), Mesothelioma (MESO), Brain Lower Grade Glioma (LGG), and Liver Hepatocellular Carcinoma (LIHC) patients and with better survival for Thymoma (THYM) and Colorectal Adenocarcinoma (COAD) patients (Figure 1A). Higher TRIM28 expression is significantly associated with worse survival for Kidney Renal Clear Cell Carcinoma (KIRC), Kidney Renal Papillary Cell Carcinoma (KIRP), LIHC, Lung Adenocarcinoma (LUAD), MESO, Adrenocortical Carcinoma (ACC), Skin Cutaneous Melanoma (SKCM), and Bladder Urothelial Carcinoma (BLCA), and with better survival for THYM, Uveal Melanoma (UVM), and Testicular Germ Cell Tumor (TGCT) patients (Figure 1B). Elevated expression of TRIM33 is significantly associated with worse survival for Uterine Corpus Endometrial Carcinoma (UCEC) patients and with better survival for COAD and KIRC patients (Figure 1C). A higher expression of TRIM66 is significantly associated with worse survival for KIRC patients and better survival for Sarcoma (SARC), Head and Neck Squamous Cell Carcinoma (HNSC), and Pancreatic Adenocarcinoma (PAAD) patients (Figure 1D).

We also looked at the frequency of alterations in TIF1 family members. Using the cBioportal data, we observed that across all tested tumor types (10,506 profiled samples in 27 solid TCGA tumor types), the frequencies of alterations (missense mutations, amplifications, deletions) in TIF1 member-encoding genes were relatively low (Figure S1A), with 2.6%, 2.2%, 1.8%, and 0.9% genetic alterations in profiled samples for TRIM24, TRIM28, TRIM33, and TRIM66, respectively. In most cancer types, the frequency of alterations did not exceed 5% for each of the tested genes (Figure S1B–E), except for TRIM24 in Ovarian Serous Cystadenocarcinoma (OV) (10.98%) and SKCM (8.05%), and TRIM28 and TRIM33 in Esophageal Carcinoma (ESCA) (5.41% and 5.41% of altered samples, respectively), suggesting that genetic alterations in TIF1 members are not of great importance in cancer development.

### 3.2. TIF1α/TRIM24 and TIF1β/TRIM28 Are Positively Associated While TIF1γ/TRIM33 and TIF1δ/TRIM66 Are Negatively Associated with Tumor Stemness

**Figure 2:**
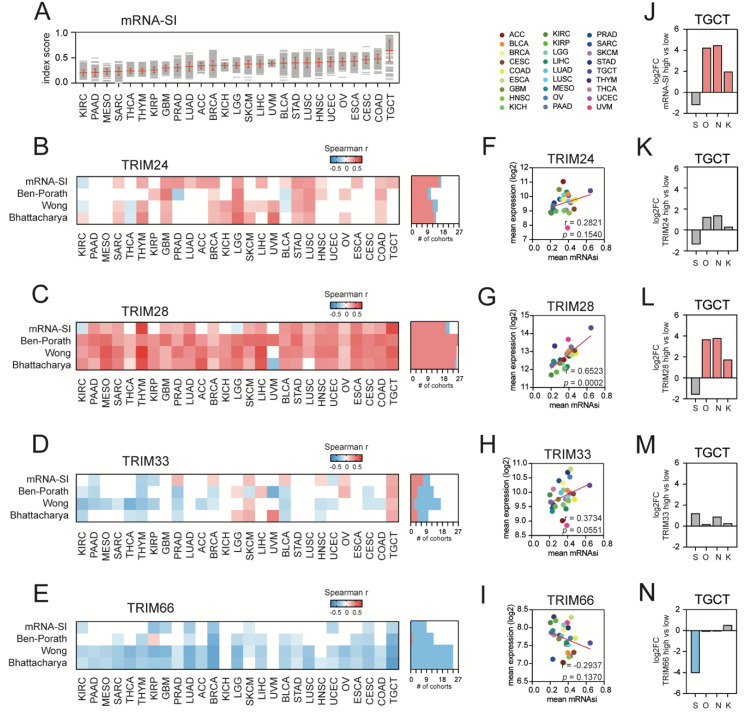
The association of the expression of TIF1 family members with cancer stemness. (**A**) The transcriptome-based stemness index (mRNA-SI) calculated for 27 TCGA solid tumor types. (**B**–**E**) The heatmap of Spearman’s correlation between (**B**) TRIM24, (**C**) TRIM28, (**D**) TRIM33, and (**E**) TRIM66 expression and distinct stemness scores (mRNA-Stemness Index, Ben-Porath ES core signature, Wong ESC core signature, Bhattacharya ESC signature). Note the strong positive association between TRIM28 expression and stemness indices and the negative association between TRIM66 expression and stemness indices across distinct tumor types. The number of TCGA cohorts characterized with either a positive (red) or negative (blue) correlation between the expression of specific TIF1 family members and tested stemness scores/signatures are shown. (**F**–**I**) The Spearman’s correlation between the mean TRIM24 (**F**), TRIM28 (**G**), TRIM33 (**H**), or TRIM66 (**I**) expression level and the average mRNA-SI score across 27 solid tumor types. (**J**) mRNA-SI^HIGH^ TGCT tumors express significantly higher levels of stemness transcription factors Octamer-binding transcription factor 4 (OCT-3/4), Nanog Homeobox (Nanog), and Kruppel Like Factor 4 (KLF4). (**K**–**N**) Among other TIF1 members, only TRIM28^HIGH^ TGCT tumors express significantly higher levels of OCT-3/4, Nanog, and KLF4. S–SOX2 (SRY (sex determining region Y)-box 2), O–OCT-3/4, N–NANOG, and K–KLF4. Red—significant upregulation; blue—significant downregulation; grey—no statistical significance (FDR > 1%).

As previously reported, TCGA solid tumors exhibit different levels of stemness features (Figure 2A) [7]. Here, we determined the correlation between the expression of TIF1 family members and cancer stemness, which was evaluated with four distinct scores/signatures: mRNA-SI [7], Ben-Porath Embryonic Stem Cell (ESC) core signature [4], Wong ESC core signature [5], and Bhattacharya ESC signature [6]. For TRIM24 and TRIM28 expression, we mostly observed positive Spearman’s correlation with stemness indices (Figure 2B,C), while for TRIM33 and TRIM66 expression, the correlation with stemness indices was primarily negative (Figure 2D,E).

Moreover, we compared the average mRNA-SI value with the mean expression of TIF1 family members across TCGA tumor types (Figure 2F–I) and observed a significant positive correlation only for TRIM28. As presented in Figure 1G, the TCGA tumor types with high average mRNA-SI scores were expressing significantly higher levels of TRIM28 (r = 0.6523, *p*-value = 0.0002).

Next, we compared the level of the top recognized stemness transcription factors, namely SRY (sex determining region Y)-box 2 (SOX2), Octamer-binding transcription factor 4 (OCT-3/4), Nanog Homeobox (Nanog), and Kruppel Like Factor 4 (KLF4), in highly stem-like testicular germ cell tumors (TGCT) divided into high and low mRNA-SI cohorts and observed a significant upregulation of OCT-3/4, Nanog, and KLF4 in the mRNA-SI^HIGH^ phenotype (Figure 2J). Similarly, using the average TIF1 expression as a cut-off, we discriminated high and low TIF1-expressing TGCT cohorts and searched for stemness transcription factors among all differentially expressed genes (Figure 2K–N). We observed that only in TRIM28^HIGH^ TGCT tumors, the expression of OCT-3/4, Nanog, and KLF4 is significantly upregulated when compared to the TRIM28^LOW^ subgroup.

### 3.3. Higher Grade Tumors Exhibit a Higher Level of mRNA-SI and Elevated TIF1β/TRIM28 Expression

**Figure 3:**
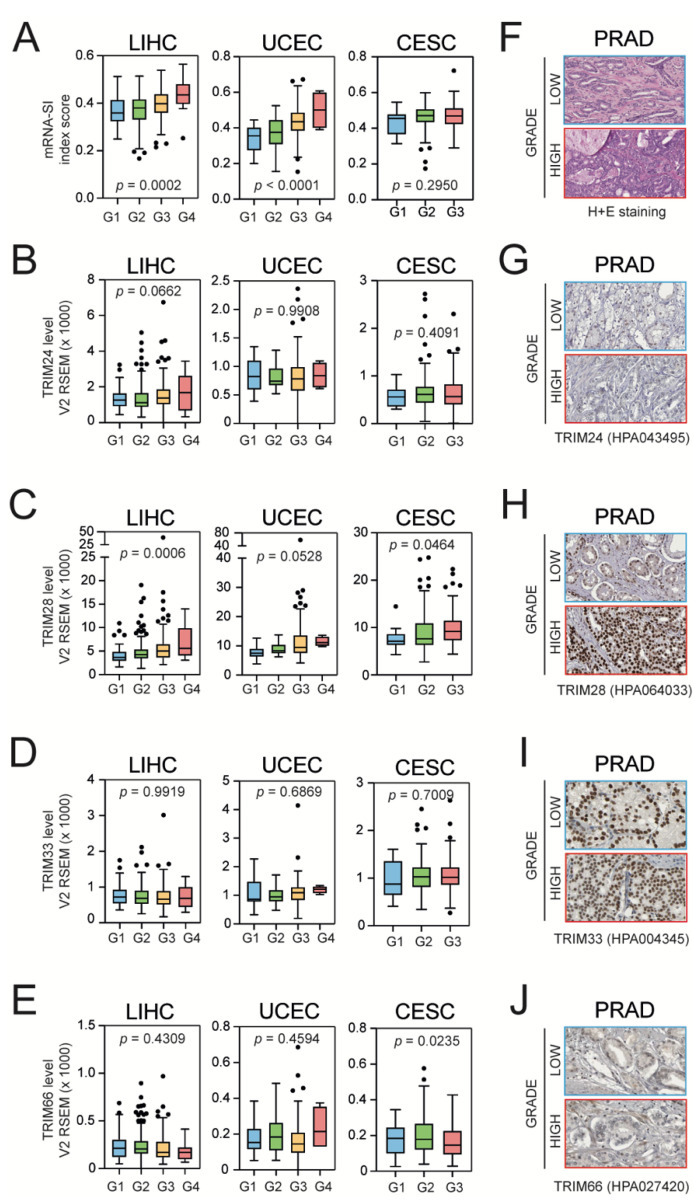
The level of mRNA-SI and TIF1 expression in lower and higher grade tumors. (**A**) Higher grade tumors exhibit higher mRNA-SI values as presented for LIHC (left panel), UCEC (middle panel), and CESC (right panel). G1–G4—grade 1–4 (the “neoplasm histologic grade” feature from TCGA data). (**B**–**E**) Among the other TIF1 family members, the expression of TRIM28 is significantly elevated in higher grade tumors. (**F**) Hematoxylin–eosin staining of lower and higher grade PRAD tumors. (**G**–**J**) Representative immunohistochemistry staining for TRIM24 (**G**), TRIM28 (**H**), TRIM33 (**I**), and TRIM66 (**J**) of lower and higher grade PRAD tumors (from www.proteinatlas.org, accessed on 10 August 2020).

As de-differentiated tumors clearly exhibit stemness characteristics, we analyzed the association between tumor grade and mRNA-SI score [7]. Indeed, we observed higher mRNA-SI levels in higher grade tumors (Figure 3A). We further analyzed the level of TIF1 family members in relation to the tumor grade using transcriptomic (Figure 3B–E) data and observed significantly higher TRIM28 expression in de-differentiated tumors (Figure 3C), while the level of other TIF1 members was relatively unchanged.

Although statistical significance was not reached, the level of TRIM28 protein in the immunohistochemistry staining of PRAD tumors (from the Human Protein Atlas [43]) seems to be elevated in higher grade tumors when compared to more differentiated tumors (in contrast to other TIF1 family members, which seem to be evenly expressed in both types). Altogether, this strongly supports the previously reported association between TRIM28 and cancer stemness [20,28,54].

### 3.4. TRIM28-Associated Gene Expression Profiles Are Significantly Enriched with the Targets for the c-Myc Transcription Factor That Mirrors the Enrichment of mRNA-SI Gene Signature with the “Hallmarks Of Cancer” Terms

**Figure 4:**
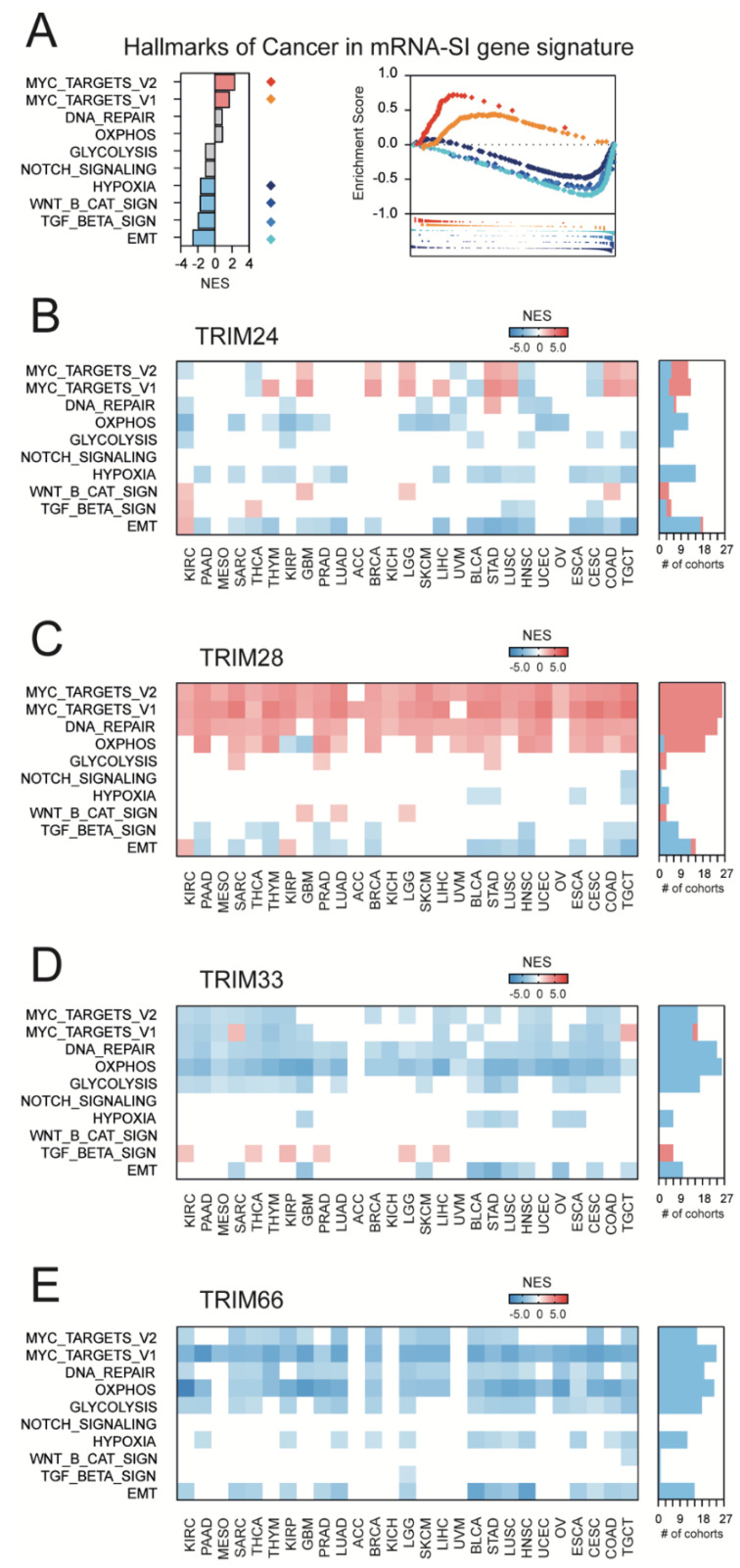
The hallmarks of cancer in mRNA-SI and TIF1-associated gene signatures. (**A**) The targets for c-Myc transcription factors are enriched, while the genes associated with hypoxia, Wnt/β-catenin signaling, Tumor Growth Factor-β (TGF-β) signaling, and Epithelial-Mesenchymal Transition (EMT) are depleted in the mRNA-SI gene signature. NES – Normalized Enrichment Score. Red—enriched terms; blue—depleted terms; grey—no statistical significance (FDR >1%). (**B**–**E**) The transcriptome profiles associated with TRIM24 (**B**), TRIM28 (**C**), TRIM33 (**D**), or TRIM66 (**E**) gene expression are differentially enriched or depleted with hallmarks of cancer terms, and TRIM28-associated transcriptome profiles are consequently enriched, while TRIM66-associated transcriptome profiles are consequently depleted with the targets for c-Myc transcription factor.

In their work, Malta et al. [7] demonstrated the significant enrichment of the mRNA-SI gene signature with targets for c-Myc (MYC Proto-Oncogene) transcription factors, followed by the substantial depletion of hypoxia, Wnt/β-catenin signaling, Tumor Growth Factor-β (TGF-β) signaling, and Epithelial-Mesenchymal Transition (EMT) (Figure 4A). Therefore, to compare the mRNA-SI gene signature with TIF1-associated gene signatures, we analyzed the presence of markers engaged in these “hallmarks of cancer” in TIF1-associated transcriptome profiles across TCGA tumors.

Firstly, we defined the TIF1-related transcription profiles in all tested TCGA solid tumor types. The number of genes that are significantly correlated with the expression of TRIM24, TRIM28, TRIM33, or TRIM66 are presented in Figure S2. Then, we used gene set enrichment analysis (GSEA) to verify whether the TIF1-associated transcription profiles are enriched or depleted with markers of “hallmarks of cancer” terms. As presented in Figure 4, only TRIM28-associated transcriptome profiles are significantly enriched with targets for the c-Myc transcription factor across most TCGA tumor types, which strongly reflects the mRNA-SI gene signature enrichment results. In contrast to TRIM24, the expression of TRIM28 positively correlates with MYC expression in several cancer types (Figure S3A–C). On the other hand, TRIM66-associated transcriptome profiles are substantially depleted of targets for the c-Myc transcription factor, which reflects a negative correlation between TRIM66 expression and cancer stemness as assessed with stemness scores/signatures (Figure 2E). Surprisingly, the expression of TRIM66 barely correlates with the level of MYC (Figure S3) in tested solid tumor types.

### 3.5. TRIM28-Associated Transcriptome Profiles Are Significantly Enriched, while TRIM66-Associated Transcriptome Profiles Are Significantly Depleted with Stemness-Associated Gene Signatures across Solid TCGA Tumor Types

**Figure 5:**
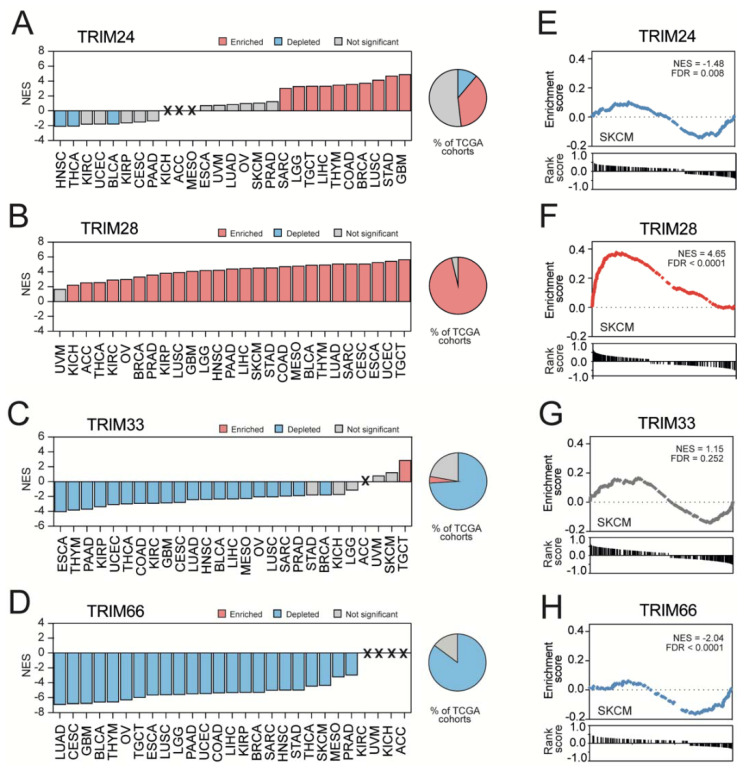
Stemness signature enrichment in transcriptome profiles associated with the expression of TIF1 family members. (**A**–**D**) The Gene Set Enrichment Analysis (GSEA) using significantly correlated genes for (**A**) TRIM24, (B) TRIM28, (**C**) TRIM33, or (**D**) TRIM66 expression was performed with the stemness signature (Wong_ESC_Core) as a reference. Bar—normalized enrichment score (NES); X—gene sets that did not reach the size threshold (at least 15 genes). Blue and red denote a significant depletion or significant enrichment, respectively. (**E**,**F**) The GSEA using transcriptome profiles associated with the expression of (**E**) TRIM24, (**F**) TRIM28, (**G**) TRIM33, and (**H**) TRIM66 in SKCM revealed significant enrichment with the Kim_Myc_targets gene signature. The GSEA results for the Kim_Myc_targets gene signature enrichment in other tumor types are shown in Figure S3.

Using GSEA, we further compared the TIF1-associated transcriptome profiles with a priori defined stemness-associated gene signatures [44]. As presented in Figure 5A, TRIM24-associated transcription profiles were significantly enriched with stem cell markers (Wong ESC core gene set) in only 10 tumor types, and the normalized enrichment score (NES) for this signature ranged from 3.15 (SARC) to 4.99 (GBM). On the other hand, TRIM24-associated transcription profiles were significantly depleted, with stem cell markers in HNSC (NES = −2.20), Thyroid Carcinoma (THCA) (NES = −2.18), and BLCA (NES = −1.90), which partially reflected the negative correlation of TRIM24 expression with stemness scores (Figure 2B).

Furthermore, we observed a significant enrichment of TRIM28-associated transcription profiles with stem cell markers in 26 out of 27 tested tumor types (Figure 5B). Except for UVM, the normalized enrichment score for stemness signature in TRIM28-associated transcription profiles ranged from 2.32 (KICH) up to 5.74 (TGCT).

As for TRIM33, the associated transcription profiles were significantly depleted, with stem cell markers in 19 tumor types and the NES values for Wong ESC core signature ranging from −4.16 (ESCA) to −2.01 (PRAD) (Figure 5C). The transcription profiles associated with TRIM66 expression were significantly depleted, with stem cell markers in 23 tumor types and the normalized enrichment score ranging from −7.01 (LUAD) to −3.07 (PRAD) (Figure 5D).

These observations were further validated with an additional gene set attributed to cancer stemness— the Kim Myc targets signature [55] (Figure 5E–H and Figure S4). Across all tested tumor types, only the TRIM28-associated transcription profiles were consequently enriched with gene expression signatures representing a stem-like phenotype. On the other hand, the TRIM66-associated transcriptome profiles were consequently depleted with stemness-related markers in almost all tested tumor types.

Next, we harnessed other publicly available datasets (Table S2) [45,46,47,48,49,50,51,52,53] and observed an analogous enrichment of TRIM28-associated transcriptome profiles, followed by a significant depletion of TRIM66-associated transcriptome profiles with stemness-related genes (Figure S5). Therefore, the TRIM28 positive association and TRIM66 negative association with tumor stemness is a common phenomenon across solid tumors.

The stemness signatures used in our study were developed based on the transcriptional profiles of undifferentiated normal stem cells and were demonstrated to be very efficient in quantifying tumor stemness. To confirm that a high level of expression of TRIM28 is truly associated with a tissue-specific cancer stem cell-like phenotype, we further tested signatures developed based on the expression profiles of stem cells isolated from distinct tissues or tumor types, namely breast tissue and glioma cancers [56,57,58]. We observed significant positive correlations for TRIM28 and the upregulated genes in stem cell populations, followed by significant negative correlations with the downregulated genes in stem cells, regardless of the tested signature (Figure S6A,B). Moreover, the GSEA of TRIM28-asssociated transcriptome profiles of 27 solid tumor types revealed significant enrichment of the gene expression profile of cancer stem cells previously discovered by Pece et al. [58] (Figure S6C), while for TRIM66-associated transcriptome profiles, the results were opposite (Figure S6D) regardless of the tumor type. This strongly supports our claim that TRIM28 is positively associated, while TRIM66 is negatively associated with cancer stemness.

### 3.6. Stemness-High Tumors Are Significantly Associated with a Worse Prognosis, and TRIM28 High Expression May Predict a “Stemness-High” Phenotype

The association between tumor stemness and adverse outcomes for some tumor types has been previously suggested, although not examined in detail. Here, we discriminated stemness-low and stemness-high patients in each tumor type using four distinct stemness scores/signatures and observed that in several tumor types, higher tumor stemness is significantly associated with worse patient survival (Figure S6). Precisely, using the average mRNA-SI value as a cut-off, we observed substantially worse outcomes for patients from stemness-high cohorts in LUAD, SKCM, PAAD, KIRC, KIRP, ACC, and SARC. Surprisingly, in four tumor types (MESO, LGG, BLCA, and STAD), when discriminated against with the mRNA-SI, higher stemness was associated with better survival (Figure S7A). Using the average Ben-Porath stemness signature value as a cut-off, we observed a significantly worse survival of patients from stemness-high cohorts in LUAD, SKCM, PAAD, KIRC, KIRP, ACC, SARC, MESO, and LGG (Figure S7B). For the Wong stemness signature, we observed significantly worse outcomes for patients from stemness-high cohorts in LUAD, SKCM, PAAD, KIRC, KIRP, ACC, MESO, BRCA, KICH, LGG, and LIHC (Figure S7C). When using the Bhattacharya stemness signature as a discriminator, we observed a significantly worse survival of patients from stemness-high cohorts in LUAD, SKCM, PAAD, KIRC, KIRP, ACC, SARC, MESO, LGG, LIHC, and HNSC (Figure S7D).

Due to the abovementioned discrepancies, we used all tested stemness scores/signatures simultaneously to unequivocally define the stemness-high cohorts in each of the tested solid tumor types (all-score stemness^HIGH^, see Material and Methods section). Note that for KICH, KIRC, KIRP, LGG, PRAD, and THCA, the mRNA-SI score was excluded, leading to a three-score stemness classifier (Figure S8).

**Figure 6:**
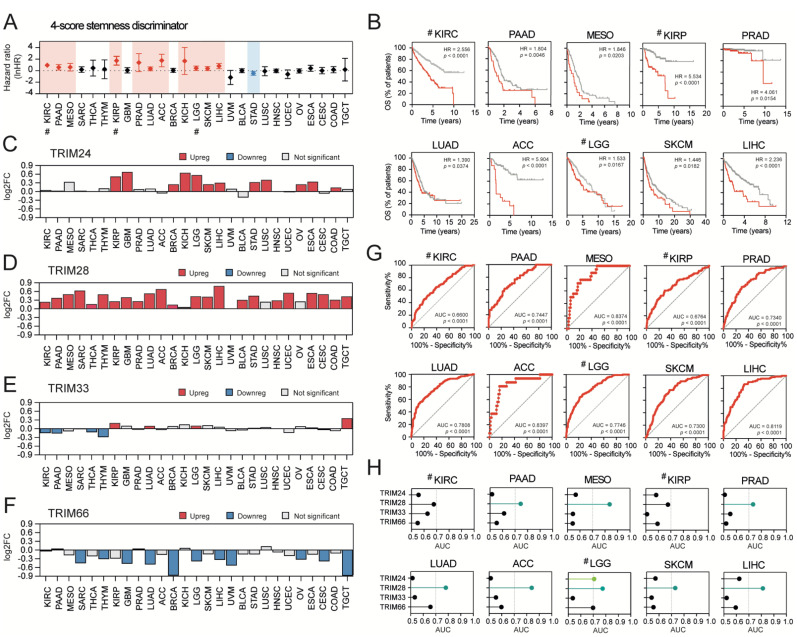
Stemness high tumors are associated with a worse prognosis, and TRIM28 high expression may predict stemness high phenotype. (**A**) The hazard ratio (lnHR) of death of 4-score stemness^HIGH^ patients across 27 TCGA tumor types (# - for KIRC, KIRP and LGG, the 3-score classifier was used). (**B**) Kaplan–Meier overall survival (OS) curves for 10 tumor types separated into stemness^HIGH^ (red) or not-stemness^HIGH^ (grey) cohorts. (**C**–**F**) The expression of (**C**) TRIM24, (**D**) TRIM28, (**E**) TRIM33, and (**F**) TRIM66 in stemness^HIGH^ tumors. Log2-normalized fold change (log2FC) between the stemness^HIGH^ and other tumors is presented. (**G**) The diagnostic value of the TRIM28 expression in predicting the stemness^HIGH^ phenotype of 10 distinct TCGA cohorts. The area under the curve (AUC) was calculated for the Receiver Operating Characteristics (ROC) curve. (**H**) Similarly, the diagnostic value for other TIF1 family members in predicting the stemness^HIGH^ phenotype across 10 TCGA tumor types is shown. The AUCs for TRIM28 exhibit the highest values.

As presented in Figure 6A,B, we observed significantly worse survival for patients in the all-score stemness^HIGH^ cohort in KIRC, PAAD, MESO, KIRP, PRAD, LUAD, ACC, KICH, SKCM, and LIHC. When we looked at the expression of TIF1 family members, only the level of TRIM28 was upregulated in all-score stemness^HIGH^ cohorts in most tumor types (except for three tumor types—OV, LUSC, and UVM) (Figure 6C–F). Moreover, the receiver operating characteristic (ROC) analysis revealed specific prognostic value for TRIM28 in predicting all-score stemness^HIGH^ cohort in contrast to other TIF1 family members (Figure 6G,H), especially in ACC (Area Under The Curve, AUC = 0.8397), MESO (AUC = 0.8374), and LIHC (AUC = 0.8119) tumors. Multivariate logistic regression analysis suggests that TRIM28 might serve as an independent marker for stemness-associated traits of these tumors (Table S3).

## 4. Discussion

Here, we analyzed the association of distinct TIF1 family members with cancer stemness across 27 types of solid tumors. Using transcriptomic data from TCGA and several other publicly available datasets, we demonstrate that: (1) among TIF family members, TRIM24 and TRIM28 are positively associated, while TRIM33 and TRIM66 are negatively associated with tumor stemness; (2) the correlation between the TRIM28^HIGH^ phenotype and cancer stemness is very robust and universal regardless of the tumor type; (3) higher-grade tumors that exhibit stem cell-like traits express higher levels of TRIM28; (4) the transcriptome profiles of TRIM28^HIGH^ solid tumors are significantly enriched with stem cell markers; (5) TRIM66-associated transcriptome profiles are significantly depleted with stem cell markers; and (6) among other TIF1 family members, only TRIM28 might serve as a marker of cancer stemness features.

We have previously shown that high TRIM28 expression is strictly related to the stem cell-like phenotype of breast cancer [20] and melanomas [28]. TRIM28 is also essential for keeping normal stem cells in their pluripotent state, at least partially by repressing the genes associated with differentiation and inducing the expression of stemness markers [26,27,54,59]. TRIM28 serves as an epigenetic barrier to induced reprogramming and the downregulation of TRIM28 facilitates the rapid acquisition of a stem-like phenotype upon exogenous expression of Yamanaka’s reprogramming factors [60].

Here, we reported for the first time that the association between high TRIM28 expression and an enriched stem cell-like phenotype is a common phenomenon across solid tumors. TRIM28 is significantly upregulated in less differentiated tumors that exhibit stem cell properties, especially stemness-related transcriptome profiles. The mean expression of TRIM28 correlates significantly with the mean stemness index (mRNA-SI) across TCGA solid tumor types, suggesting that the more de-differentiated a tumor is, the higher the expression of TRIM28.

The upregulation of TRIM28 in several types of cancers has previously been demonstrated [19,20,21,22,23,24,25], although it was not linked with the cancer stem cell-like phenotype. Here, we present that TRIM28 is closely associated with tumor de-differentiation in TCGA solid tumors, and for several tumor types, TRIM28 possesses potential diagnostic value in predicting the stemness high phenotype (which corresponds to worse patient survival).

To date, several potential modes of action for TRIM28 have been proposed, including TRIM28 acting as a transcriptional co-repressor that switches off the expression of differentiating genes [59] while enhancing the expression of stem cell markers [54]. In TCGA data, the TRIM28^HIGH^ tumors were significantly enriched with a previously defined stemness gene signature that represents c-Myc target genes [55]. TRIM28 directly interacts with c-Myc [61]. As c-Myc is sufficient to induce a cancer stem cell phenotype in epithelial cancers [5,62], it is possible that, at least partially, the cancer stem cell-like phenotype of TRIM28^HIGH^ tumors results from significant c-Myc activation. On the other hand, TRIM28 acts as an E3 ubiquitin ligase (through the RING finger) that forms a complex with various proteins to target them for ubiquitination/degradation [63], which might subsequently result in stemness maintenance [64]. Among TRIM28 ubiquitination targets, the 5’ AMP-activated protein kinase (AMPK), which regulates the “metabolic switch” in cancer cells, is known for impairing cancer stem-like phenotype acquisition [65,66]. Therefore, further studies are needed to clarify the exact role of TRIM28 in facilitating a stem cell-like phenotype across distinct solid tumor types.

As for TRIM24, its involvement in stemness regulation was previously proposed by Rafiee et al. [18]. In mouse embryonic stem cells, Trim24 converges with Oct-3/4, Sox2, and Nanog on multiple enhancers and suppresses the expression of developmental genes while activating cell cycle genes. Trim24 overexpression also significantly improved the efficiency of somatic cell reprogramming, uncovering its direct engagement in establishing pluripotency [18]. However, the association between TRIM24 and cancer stemness remains largely unresolved. Lv et al. [14] demonstrated that TRIM24 is essential to mediate Epidermal Growth Factor Receptor (EGFR)-driven glioma stem cell self-renewal. TRIM24 was proposed as an oncogenic transcriptional co-activator of Signal Transducer and Activator of Transcription 3 (STAT3), that in response to EGFR, leads to stabilized STAT3–chromatin interactions and the subsequent activation of STAT3 downstream signaling. This STAT3/TRIM24/ID1 axis mediates glioma stem cell proliferation and self-renewal. TRIM24 was also demonstrated to promote stemness and the invasiveness of GBM through direct activation of SOX2 expression [15]. In our data, TRIM24 high expression correlated with cancer stemness in both lower-grade gliomas (LGG) and glioblastomas (GBM), as well as several other cancer types (BRCA, LUSC, STAD, and COAD). It would be of interest to verify whether the abovementioned TRIM24/STAT3/ID1 axis mediates the EGFR-driven stem cell self-renewal in other types of tumors. Whether TRIM24 and TRIM28 can co-operate in cancer stemness regulation should also be addressed, as in several tumor types (i.e., BRCA, LUSC, or STAD), their expression, as well as their associated transcriptome profiles, are simultaneously correlated or enriched with stem cell markers and both TRIM24 and TRIM28 are direct regulators of core stem cell transcription factors in normal stem cells [10,18].

Previously, the engagement of TRIM33 protein in the regulation of stem cell phenotype has been presented in a normal embryonic stem cell population [35,36]. In contrast to TRIM24 and TRIM28, TRIM33 seems to be a positive regulator of stem cell differentiation. As presented by Massague et al. [35], the loss of TRIM33 expression does not affect stem cell self-renewal, but it impairs the differentiation process. Therefore, it is not surprising that TRIM33 expression negatively correlates with cancer stemness, at least in several tumor types. We report, for the first time, a significant depletion of TRIM33-associated transcriptome profiles with stemness-related genes in most TCGA tumor types. However, further studies are needed to determine whether TRIM33 acts as a transcriptional co-repressor of the expression of stemness markers, resulting in the loss of cancer stem cell self-renewal, or rather enhances the differentiation processes through the transcriptional activation of differentiating genes.

The last member of the TIF1 family, TRIM66, is the least well known, with only several papers demonstrating its engagement in tumorigenesis, and neither of these papers depicted the TRIM66–cancer stemness association [37,38,39]. According to the literature, TRIM66 is critical for DNA damage repair in embryonic stem cells, safeguarding their genomic stability [67]. However, its role in stem cell self-renewal was not tested in detail and remains unresolved. Here, we demonstrate a significant negative association between TRIM66 expression and cancer stemness across most solid TCGA tumors. As for TRIM33, the transcriptome profiles associated with high TRIM66 expression are significantly depleted with stem cell markers. This phenomenon is universal regardless of the tumor type in solid TCGA tumors. However, it remains unresolved whether TRIM66 serves as a transcriptional co-repressor for stemness markers in solid cancers.

## 5. Conclusions

To conclude, our work here demonstrates that TIF1 family members exhibit distinct expression patterns in stem cell-like tumors, despite their structural and functional similarity. TRIM24 and TRIM28 are generally positively associated, while TRIM33 and TRIM66 are mostly negatively associated with cancer stemness in solid tumors. Of note, among other TIF1 members, only the association between TRIM28 high expression and cancer stemness is very robust and universal regardless of the tumor type, with higher-grade tumors exhibiting elevated TRIM28 expression. In many tumor types, stemness-high tumors are significantly associated with a worse prognosis and among other TIF1 members, only TRIM28 might serve as a marker of cancer stemness features. However, molecular studies are needed to determine the role of TRIM28 in the acquisition or maintenance of a stem cell-like cancer phenotype across distinct types of solid tumors.

## Data Availability

All of the TCGA data used in this study are publicly available at the cBioportal database (www.cbioportal.org). Additional datasets were downloaded from the R2 Genomics Analysis and Visualization Platform (http://r2.amc.nl).

## Supplementary Materials

The following are available online at https://www.mdpi.com/2072-6694/13/7/1528/s1, Figure S1: The alteration frequencies in TIF1 family members according to the cBioportal database; Figure S2: The transcriptome profiles associated with the expression of TIF1 family members in solid tumors; Figure S3: The Spearman’s correlation between TIF1 family members and MYC expression in 27 solid tumor types; Figure S4: The stemness signature enrichment in transcriptome profiles associated with the expression of TIF1 family members; Figure S5: The stemness signature enrichment in transcriptome profiles associated with the expression of TIF1 family members in additional datasets; Figure S6: The association between TIF1 expression and cancer stem cell-specific gene expression profiles in solid tumors. Figure S7: The hazard ratio (lnHR) of death of stemness^HIGH^ patients across 27 TCGA tumor types; Figure S8: The relationship between four distinct stemness scores across 27 TCGA tumor types; Table S1: The average expression of TIF1 members in 27 solid TCGA tumor types; Table S2: Additional datasets used in this study; Table S3: Multivariate logistic regression analysis for predictive markers of the stemness^HIGH^ tumor phenotype.
