# Supplementary material for: The Association between TIF1 Family Members and Cancer Stemness in Solid Tumors
Source: Cancers (Basel). 2021 Mar 26;13(7):1528. doi: 10.3390/cancers13071528 (PMC8061774; doi:10.3390/cancers13071528)
Supplement: Supplementary file 1 [file cancers-13-01528-s001.zip › Figures S1-S8_rev.docx]

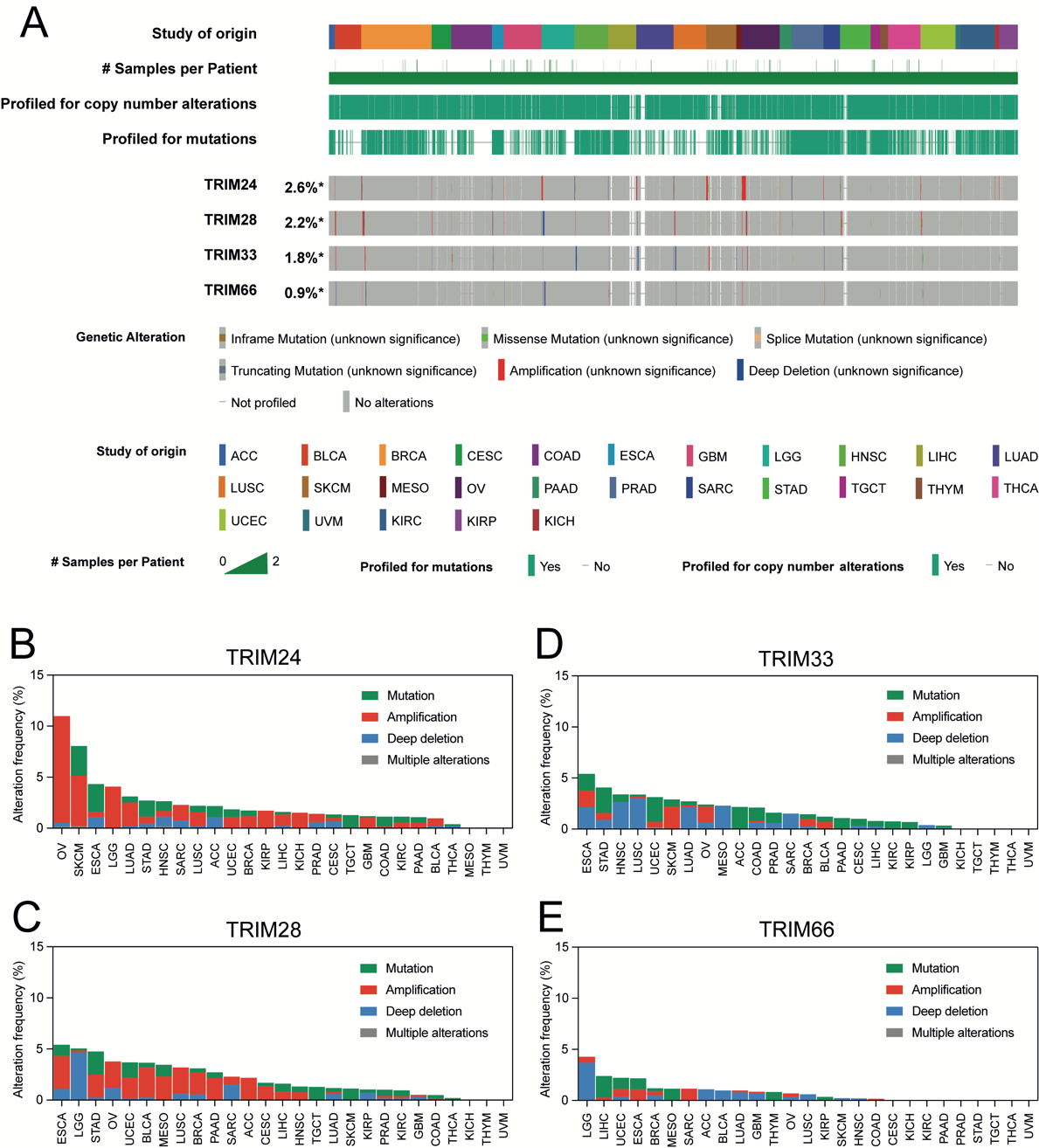


**Figure S1.** The alteration frequencies in TIF1 family members according to cBioportal database. (A) TIF1 members are rarely mutated in solid tumors. TCGA tumors are color-coded. The types of genetic alterations (inframe, missense, splice, truncating mutation, amplification, or deep deletion) are described in the legend. (B-E) The frequencies of alterations in TRIM24 (B), TRIM28 (C), TRIM33 (D), and TRIM66 (E) across 27 solid TCGA tumor types. Green - Inframe, missense, splice, or truncating mutations; Red – amplifications; Blue - deep deletions; Grey – multiple alterations. Data are shown as the proportion of profiled samples in each tumor type.


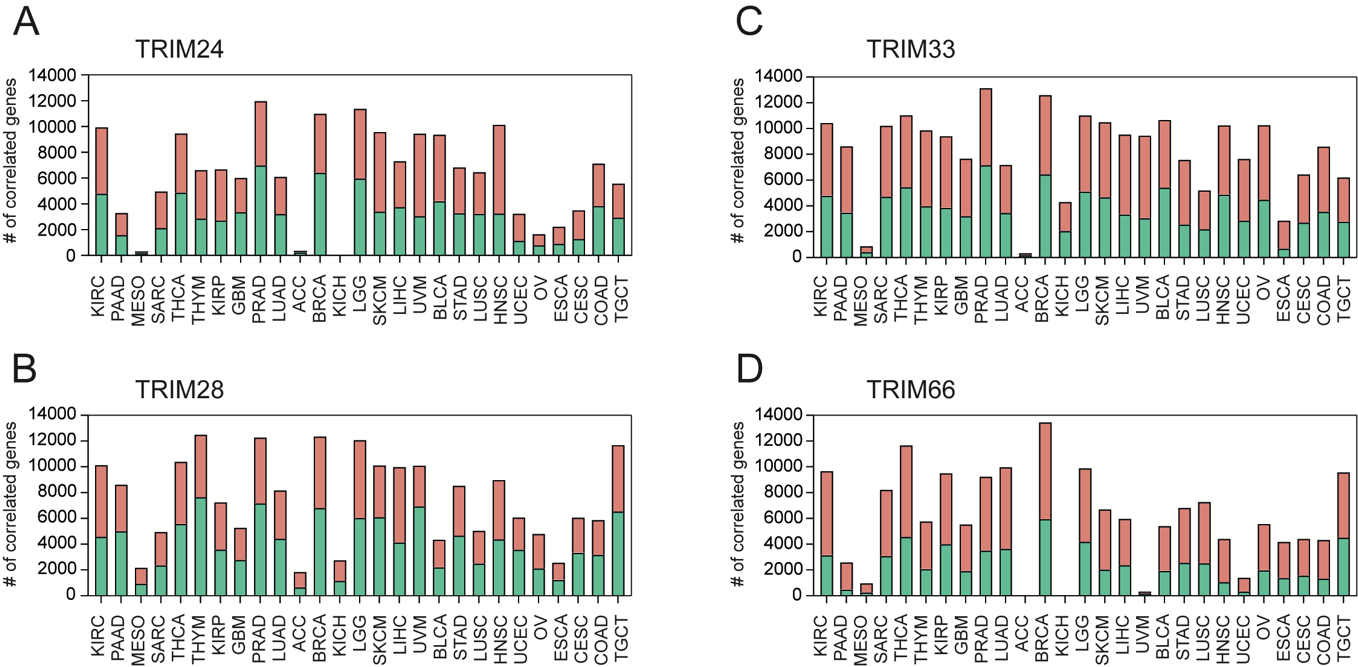


**Figure S2.** The transcriptome profiles associated with TIF1 family members’ expression in solid tumors. The number of genes significantly correlated (adj. p-val < 0.01) with the expression of (A) TRIM24, (B) TRIM28, (C) TRIM33, or (D) TRIM66 in each tumor type. Green and red denote negative and positive correlation, respectively.


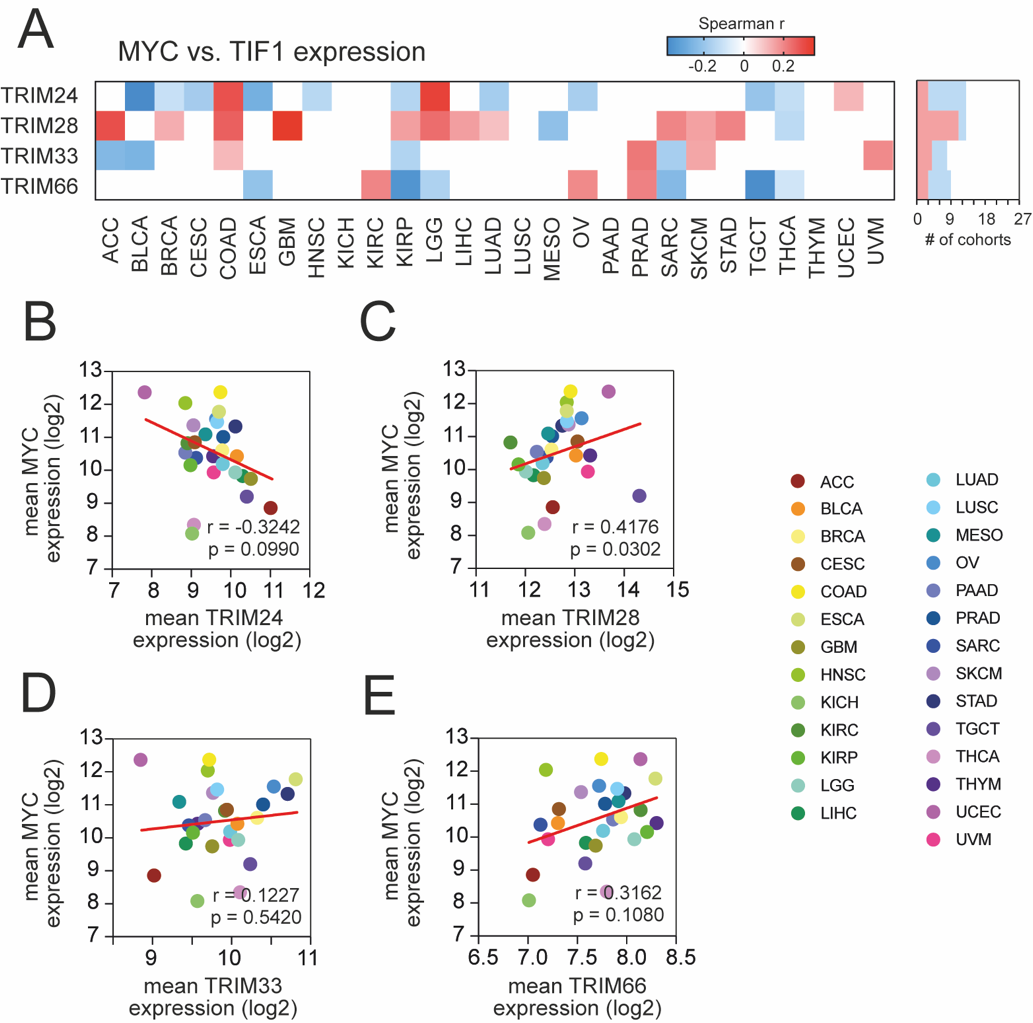


**Figure S3.** The Spearman correlation between TIF1 family members and MYC expression in 27 solid tumor types. (A) The heatmap of Spearman correlation calculated for each tumor type separately. Only statistically significant (p < 0.05) results are shown. The number of TCGA cohorts characterized with either positive (red) or negative (blue) correlation between the expression of specific TIF1 family members MYC are shown. (B-E) The Spearman correlation between the mean TRIM24 (B), TRIM28 (C), TRIM33 (D), or TRIM66 (E) expression level and the average MYC level across 27 solid tumor types.


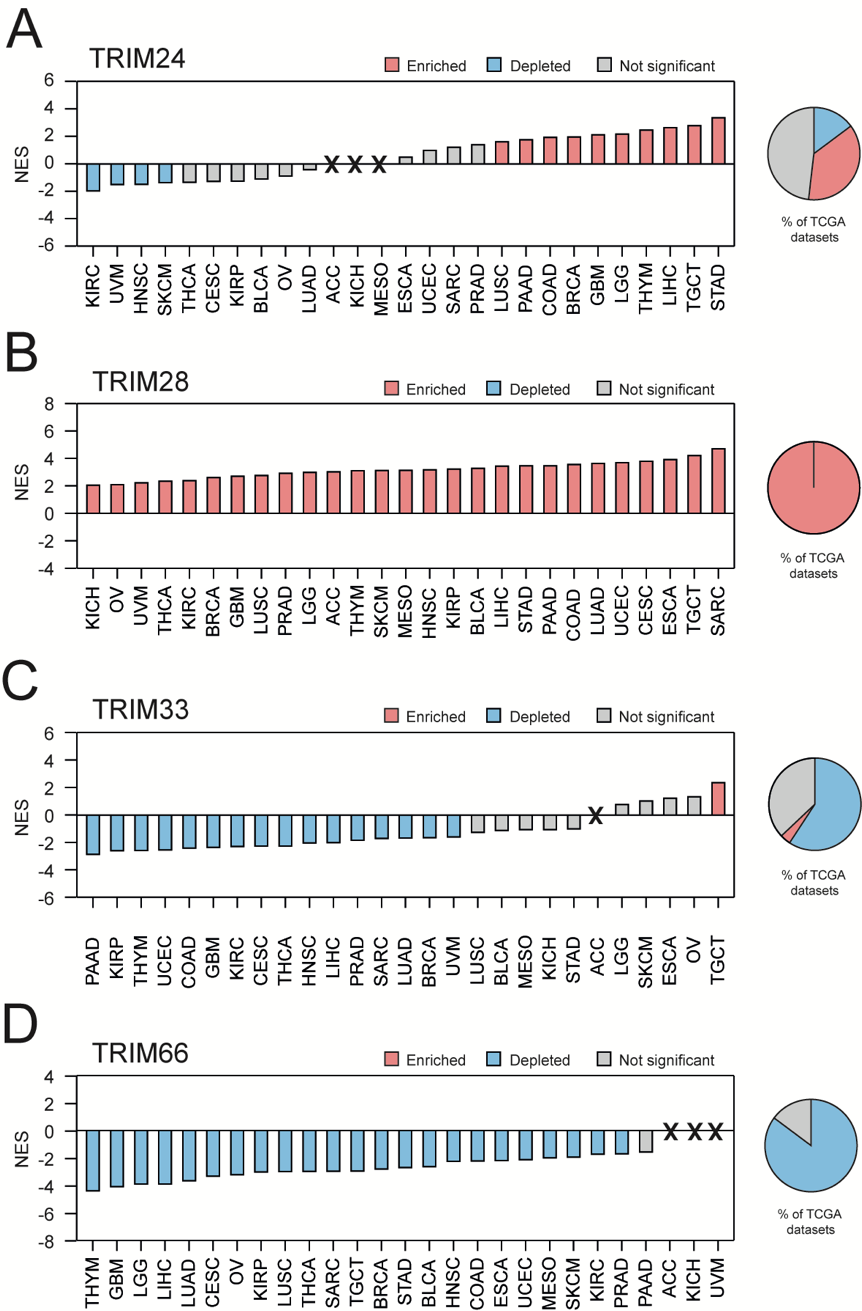


**Figure S4.** The stemness signature enrichment in transcriptome profiles associated with TIF1 family members’ expression. The GSEA using significantly correlated genes to (A) TRIM24, (B) TRIM28, (C) TRIM33, or (D) TRIM66 expression was performed with the stemness signature (Kim_Myc_targets) as a reference. Blue and red denotes significant depletion or significant enrichment, respectively.


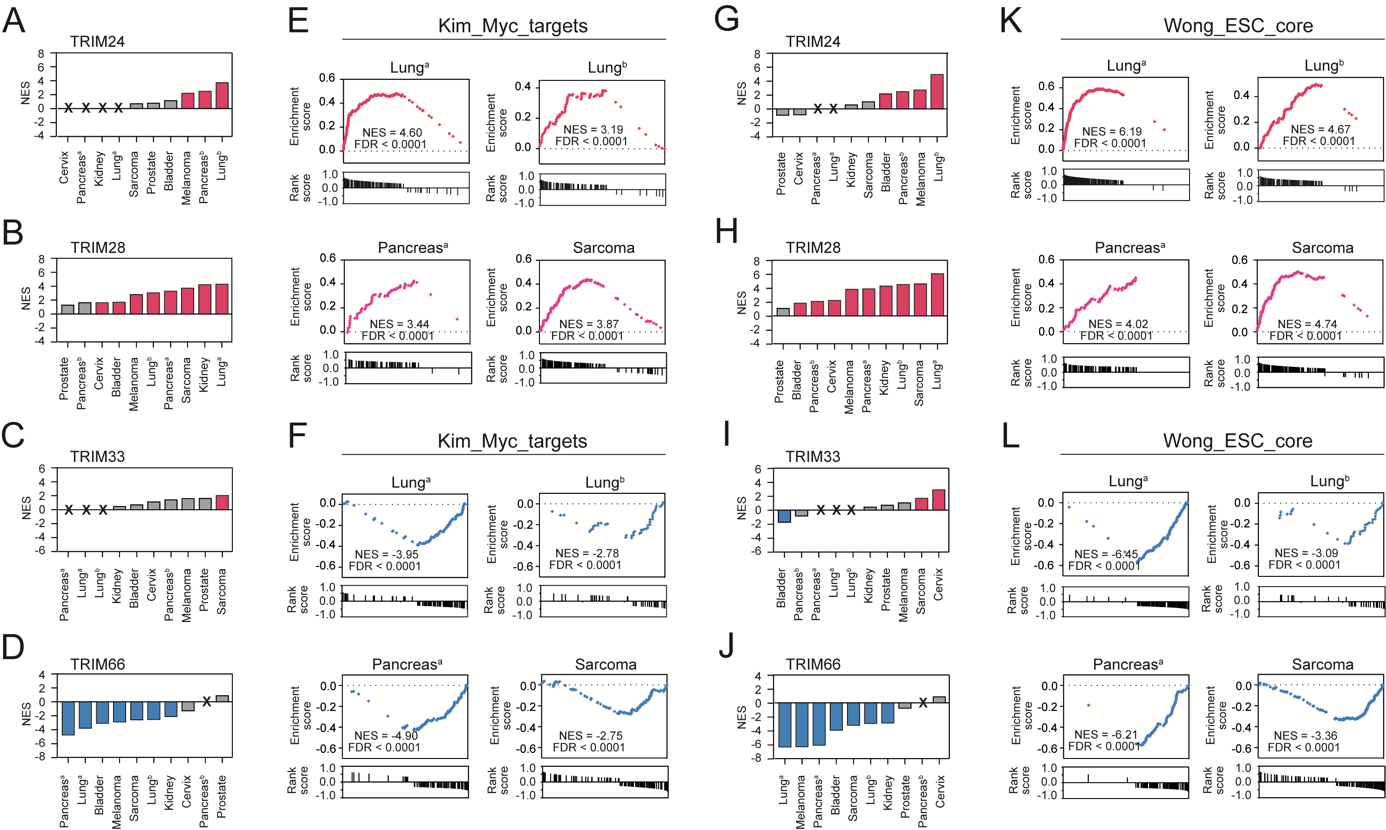


**Figure S5.** The stemness signature enrichment in transcriptome profiles associated with TIF1 family members’ expression in additional datasets. (A-D) The GSEA using significantly correlated genes to TRIM24 (A), TRIM28 (B), TRIM33 (C), and TRIM66 (D) expression in additional datasets (for further details, see Table S2) was performed with the stemness signature Kim_Myc_targes as a reference. Bar – Normalized Enrichment Score (NES), X – gene sets that did not reach the threshold of the size (at least 15 genes). Blue and red denotes significant (FDR < 1%) depletion or significant enrichment, respectively. (E-F) The enrichment plots for selected datasets from (B) and (D), respectively. (G-J) Similarly, the GSEA using significantly correlated genes to TRIM24 (G), TRIM28 (H), TRIM33 (I), and TRIM66 (J) expression in additional datasets (for further details, see Table S2) was performed with the stemness signature Wong_ESC_core as a reference. Bar – Normalized Enrichment Score (NES), X – gene sets that did not reach the threshold of the size (at least 15 genes). Blue and red denotes significant (FDR < 1%) depletion or significant enrichment, respectively. (K-L) The enrichment plots for selected datasets from (H) and (J), respectively.


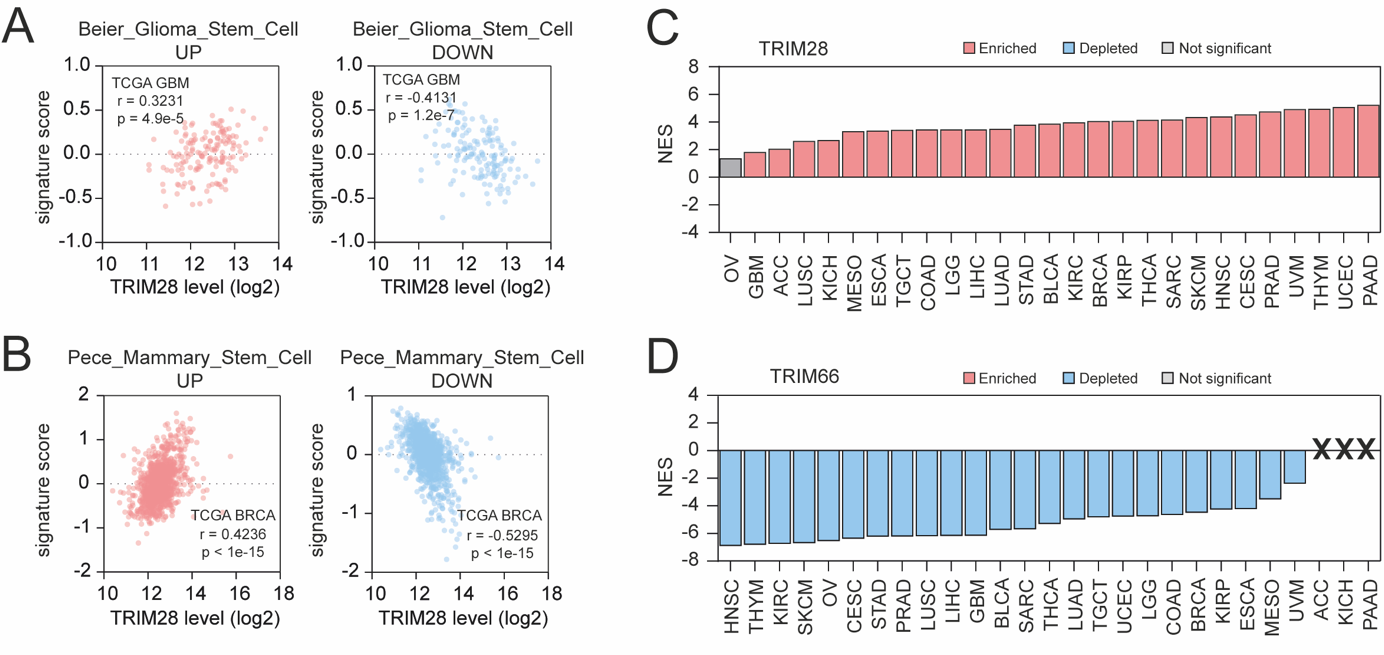


**Figure S6.** The association between TIF1 expression and cancer stem cell-specific gene expression profiles in solid tumors. (A) The Spearman correlation between TRIM28 expression and “Beier Glioma Stem Cell upregulated genes” signature (left) or “Beier Glioma Stem Cell downregulated genes” (right) in TCGA GBM cohort. (B) The Spearman correlation between TRIM28 expression and “Pece Mammary Stem Cell upregulated genes” signature (left) or “Pece Mammary Stem Cell downregulated genes” (right) in TCGA BRCA cohort. (C-D) The GSEA using significantly correlated genes to (C) TRIM28 or (D) TRIM66 expression in 27 TCGA solid tumor types performed with the cancer stem cell signature from [58] as a reference. Bar – Normalized Enrichment Score (NES), X – gene sets that did not reach the threshold of the size (at least 15 genes). Light blue and light red denotes significant (FDR < 1%) depletion or significant enrichment, respectively.


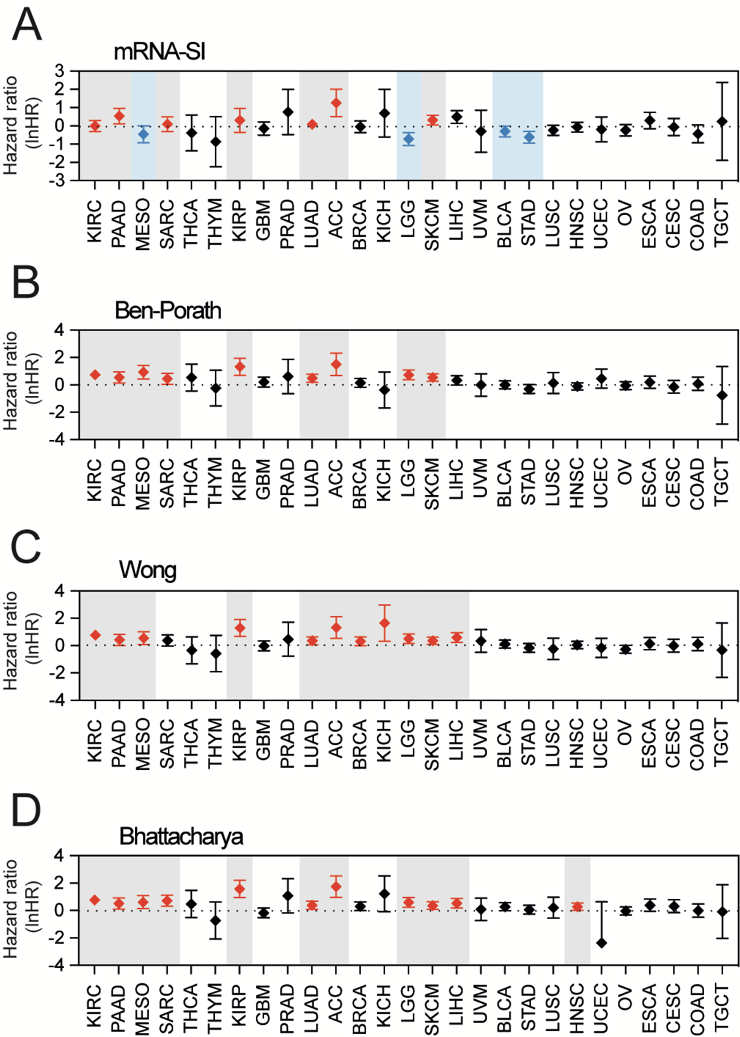


**Figure S7.** The hazard ratio (lnHR) of death of stemness^HIGH^ patients across 27 TCGA tumor types. The stemness^HIGH^ cohort was discriminated with (A) the mRNA-SI, (B) the Ben-Porath signature, (C) the Wong signature, or (D) the Bhattacharya signature score, respectively, using the mean value as a cut-off.


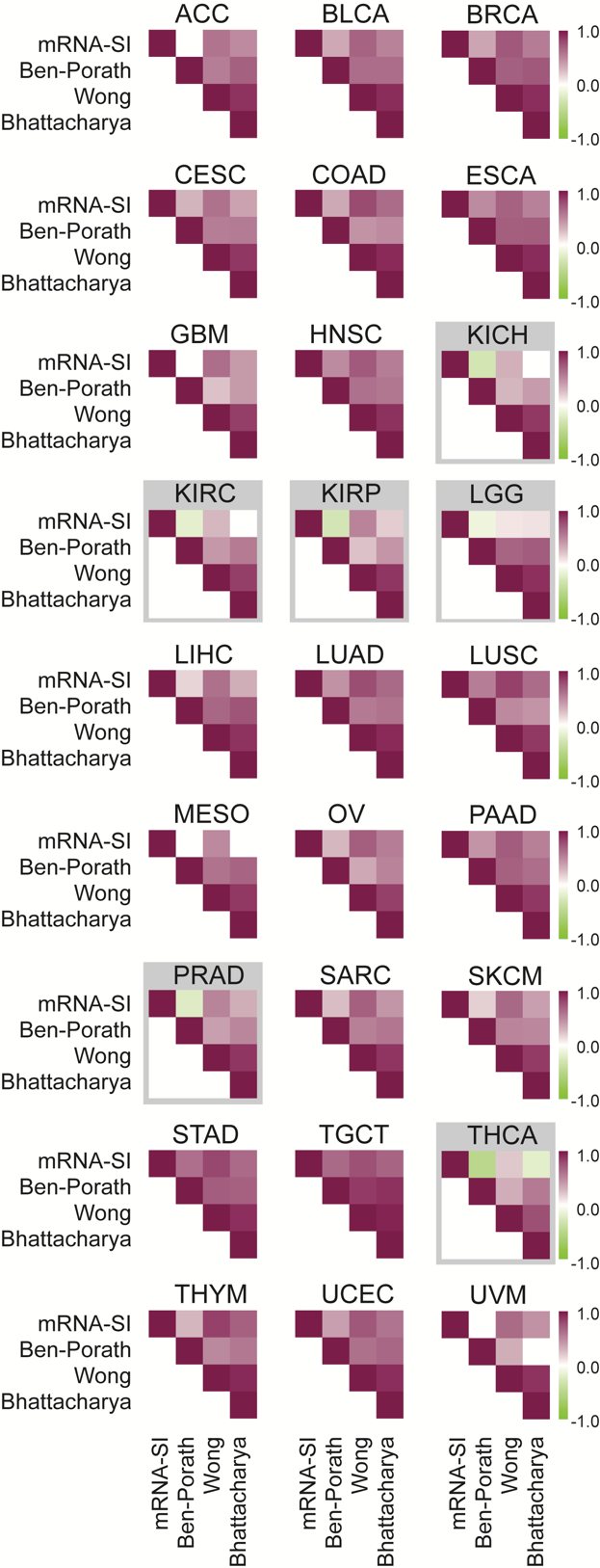


**Figure S8.** The relationship between four distinct stemness scores across 27 TCGA tumor types. Tumor types marked with grey exhibit negative correlation between at least two of the tested stemness scores.
